# Metabolic Syndrome Among Adults in New York City, 2004 New York City Health and Nutrition Examination Survey

**Published:** 2011-12-15

**Authors:** Hannah T. Jordan, Bahman P. Tabaei, Sonia Y. Angell, Shadi Chamany, Bonnie Kerker, Denis Nash

**Affiliations:** New York City Department of Health and Mental Hygiene; New York City Department of Health and Mental Hygiene, New York, New York; New York City Department of Health and Mental Hygiene, New York, New York; New York City Department of Health and Mental Hygiene, New York, New York; New York City Department of Health and Mental Hygiene, New York, New York; Columbia University Mailman School of Public Health, New York, New York

## Abstract

**Introduction:**

The objective of this study was to describe the prevalence of and factors associated with metabolic syndrome among adult New York City residents.

**Methods:**

The 2004 New York City Health and Nutrition Examination Survey was a population-based, cross-sectional study of noninstitutionalized New York City residents aged 20 years or older. We examined the prevalence of metabolic syndrome and its components as defined by the National Cholesterol Education Program's Adult Treatment Panel III revised guidelines, according to demographic subgroups and comorbid diagnoses in a probability sample of 1,263 participants. We conducted bivariable and multivariable analyses to identify factors associated with metabolic syndrome.

**Results:**

The age-adjusted prevalence of metabolic syndrome was 26.7% (95% confidence interval, 23.7%-29.8%). Prevalence was highest among Hispanics (33.9%) and lowest among whites (21.8%). Prevalence increased with age and body mass index and was higher among women (30.1%) than among men (22.9%). More than half (55.4%) of women and 33.0% of men with metabolic syndrome had only 3 metabolic abnormalities, 1 of which was abdominal obesity. The most common combination of metabolic abnormalities was abdominal obesity, elevated fasting blood glucose, and elevated blood pressure. Adjusting for other factors, higher body mass index, Asian race, and current smoking were positively associated with metabolic syndrome; alcohol use was inversely associated with metabolic syndrome among women but increased the likelihood of metabolic syndrome among men.

**Conclusion:**

Metabolic syndrome is pervasive among New York City adults, particularly women, and is associated with modifiable factors. These results identify population subgroups that could be targeted for prevention and provide a benchmark for assessing such interventions.

## Introduction

Several risk factors for cardiovascular disease (CVD), including abdominal obesity, elevated fasting blood glucose, elevated triglycerides, elevated blood pressure, and low high-density lipoprotein (HDL) cholesterol, tend to cluster in individuals (1). The simultaneous presence of 3 or more of these has been termed the metabolic syndrome (MetS). Even when these metabolic abnormalities are present at predisease levels, MetS is associated with an elevated CVD risk and with cardiovascular and all-cause mortality (2,3).

Much remains unknown about MetS, including its causes. Whether or not MetS confers greater CVD risk than the sum of its parts is also subject to debate (4). Because of these uncertainties and a lack of clinical evidence, there is no consensus on screening for MetS or on treatment of people with MetS who do not have diabetes, hypertension, or dyslipidemia. Nonetheless, people with MetS are at higher risk of CVD and may benefit from long-term follow-up and aggressive preventive care (5).

In addition to its potential to identify high-risk people, MetS is a useful indicator of population-level CVD risk. The National Health and Nutrition Examination Survey (NHANES) 2003-2006 found that 34% of US adults have MetS (6). MetS prevalence varies among racial and ethnic groups (7); other factors associated with MetS include older age, lower socioeconomic status, and smoking (7,8).

Whether NHANES reflects the epidemiology of MetS in settings with marked socioeconomic and racial/ethnic diversity is unknown. One study describing MetS among New York City adults who did not have diabetes focused on differences between Asians and whites (9). The objective of our study was to describe the prevalence of and factors associated with MetS among New York City adults among all racial/ethnic groups. We also evaluated the prevalence of MetS among participants with comorbid conditions such as diabetes and hypertension.

## Methods

The New York City (NYC) Health and Nutrition Examination Survey (NYC HANES) 2004 is described elsewhere (10). It was a population-based, cross-sectional survey of noninstitutionalized adult NYC residents. Participants gave informed consent for participation. The NYC Department of Health and Mental Hygiene (DOHMH) institutional review board approved the study.

Eligible participants were selected for the survey by using a 3-stage cluster sampling design. Between June 2 and December 19, 2004, participants completed a face-to-face, computer-assisted interview, physical examination, and biologic specimen collection, including fasting blood glucose measurements. Of 3,047 eligible NYC residents, 1,999 completed the interview and at least 1 examination component (overall response rate, 55%). We excluded pregnant women (n = 12) and people who did not complete either (or both) examination component (n = 649). Of 1,338 nonpregnant NYC HANES participants with both examination components, we then excluded people (n = 75) who lacked valid data on fasting blood glucose, triglycerides, HDL cholesterol, waist circumference, blood pressure, and prescription medication use for diabetes or hypertension. We thus included 1,263 of 1,338 (94%) participants with complete data in this analysis.

### MetS criteria

We used the MetS definition used for NHANES 2003-2006 (6). This definition incorporates medical treatment for hypertension and elevated fasting blood glucose (5) but is otherwise identical to the Third Report of the National Cholesterol Education Program (NCEP) Adult Treatment Panel (ATP III) MetS definition (11). We thus defined MetS as the presence of 3 or more of the following: abdominal obesity (waist circumference ≥88 cm in women or ≥102 cm in men); low HDL cholesterol (<50 mg/dL in women or <40 mg/dL in men); elevated triglycerides (≥150 mg/dL); elevated fasting blood glucose (≥100 mg/dL or use of oral hypoglycemic medication or insulin or both); or elevated blood pressure (at least 1 of the following: systolic ≥130 mmHg, diastolic ≥85 mmHg, or use of antihypertensive medication). We did not collect data on medication use for elevated triglycerides or low HDL cholesterol.

### Variable definitions

We recategorized self-reported race and Hispanic ethnicity as non-Hispanic white, non-Hispanic black, Hispanic, or non-Hispanic Asian; we excluded 17 people reporting "other race" from analyses on race/ethnicity. We defined people born in the United States or US territories as US-born. We defined educational levels as less than a high school degree, completion of high school or general educational development (GED) certificate, or more than a high school degree or GED. We considered people reporting any form of private health insurance to have private insurance; people reporting no private insurance but Medicare, Medicaid, or any other government coverage to have government insurance; and people reporting no health insurance to be uninsured. We categorized people who reported having no routine place of health care or receiving care at emergency departments as lacking a routine place of care. We defined smoking as current (having smoked ≥100 cigarettes in a lifetime and currently smoking every day or on some days), past (having smoked ≥100 cigarettes in a lifetime but not currently smoking), or never (having smoked <100 cigarettes in one's lifetime). We defined alcohol use as heavy (average of >2 drinks per day for men and >1 drink per day for women in the past year), moderate (less frequent alcohol use in the past year), or low/none (no drinking reported in the past year).

We defined clinical hypertension as the presence of at least 1 of the following: systolic measurement of 140 mmHg or higher, diastolic measurement of 90 mmHg or higher, or use of antihypertensive medication; diabetes as the presence of at least 1 of the following: a fasting blood glucose of more than 125 mg/dL, use of oral hypoglycemic medication or insulin, or a self-reported history of clinician-diagnosed diabetes; and high LDL cholesterol according to ATP III guidelines (12). We calculated body mass index (BMI, kg/m^2^) by using measured height and weight; we defined underweight as a BMI less than 18.5, normal weight as a BMI of 18.5 to 24.9, overweight as a BMI of 25.0 to 29.9, and obesity as a BMI 30 or higher. We obtained data on menopausal status from questionnaire responses. We combined underweight and normal weight into a single category for multivariable analyses.

### Statistical methods

Unless otherwise noted, prevalence estimates were age-adjusted to the 2000 US standard population. Among people with MetS stratified by sex, we examined metabolic abnormalities that resulted in the classification of MetS. We ranked various combinations of metabolic abnormalities from the most to the least common to examine their relative importance and the extent to which metabolic abnormalities differed by sex. We examined the prevalence of MetS among participants with and without diabetes, hypertension, high LDL cholesterol, and obesity.

We used the *t* test to determine significant differences in prevalence rates for categorical variables and calculated relative standard errors (RSEs) and 95% confidence intervals (CIs) for percentages. We considered *P* values of less than .05 to be statistically significant and estimates with an RSE of 30% or more or a denominator of 50 people or fewer to be unreliable. We used SAS version 9.1 (SAS Institute, Cary, North Carolina) with SUDAAN version 10 (Research Triangle Institute, Research Triangle Park, North Carolina) to account for the complex survey design and incorporated sample weights adjusted for differential selection probabilities and survey nonresponse to produce estimates representative of the NYC population.

We constructed multiple logistic regression models to identify factors independently associated with MetS. We entered each variable with a *P* value of .20 or less for the Wald *χ* ^2^ test in bivariable analysis into the model individually and retained characteristics associated with MetS with a *P* value of less than .05 in final models. On the basis of sex differences identified in bivariable analysis, we tested interactions between sex and race/ethnicity, smoking status, alcohol use, and BMI, and found a significant interaction between sex and alcohol use. To further explore this interaction, we examined median values for key metabolic measurements according to level of alcohol use separately for men and women. We calculated adjusted odds ratios (AORs) and 95% CIs and adjusted prevalence estimates by using predicted marginals. We assessed multicollinearity in the final model; the highest level of correlation among covariates was less than 0.20.

## Results

The final sample was 53.5% female, 39.2% white, 26.5% Hispanic, 23.3% black, and 11.0% Asian. Nearly half of participants (48.3%) were born outside the United States. Although most (54.1%) had completed more than a high school education, 39.1% had an annual family income of less than $25,000. More than half of the population was overweight (35.4%) or obese (25.7%). People with incomplete data did not differ from people with complete data in age distribution, sex, race/ethnicity, or education, but those with incomplete data were more likely to have a family income of less than $25,000 and to be overweight or obese.

### Prevalence of metabolic abnormalities

Among women, the most prevalent metabolic abnormality was abdominal obesity (64.3%) (Table 1). Elevated fasting blood glucose was the most prevalent abnormality among men (43.1%). Low HDL levels were more common among women than among men (women, 30.9% vs men, 24.2%; *t* = 2.51, *P* = .01). Only one-fourth of participants had no metabolic abnormalities, whereas nearly half had 1 or 2 abnormalities.

### Prevalence of metabolic syndrome

The overall age-adjusted prevalence of metabolic syndrome among NYC adults was 26.7% and was higher among women than among men (women, 30.1% vs men, 22.9%; *t* = 2.68, *P* = .008) (Table 2). Overall, Hispanics had a higher prevalence of MetS than whites (Hispanic, 33.9% vs white, 21.8%; *t* = 3.32, *P* = .001).

Certain behavioral factors were associated with MetS prevalence. The prevalence of MetS was higher among current smokers (33.9%) than among never-smokers (25.2%) (*t* = 2.48, *P* = .01). Prevalence was higher among women with low or no alcohol use (38.1%) than among women who drank moderately (26.1%) (*t* = −2.86, *P* = .005) or heavily (12.0%) (*t* = −4.49, *P* < .001). In contrast, prevalence was higher among men with heavy alcohol use (36.6%) than among men with low or no alcohol use (21.8%), although this difference was not significant (*t* = 1.70, *P* = .06). MetS prevalence increased steadily with BMI; no underweight participants had MetS.

Among both women and men with MetS, the most common combination of metabolic abnormalities was abdominal obesity, elevated fasting blood glucose, and elevated blood pressure (16.6% of men with MetS, 21.5% of women with MetS). More than half of women (55.4%) and 33.0% of men with MetS had only 3 metabolic abnormalities, 1 of which was abdominal obesity.

### Comorbid diagnoses

**Figure 1.**
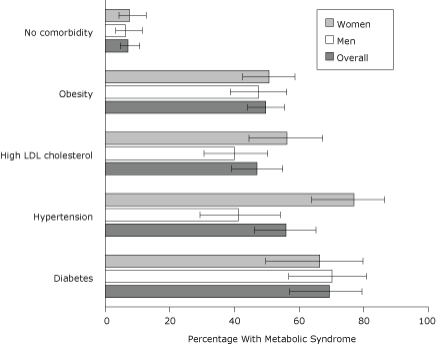
Age-adjusted prevalence of metabolic syndrome according to comorbid diagnosis, 2004 New York City Health and Nutrition Examination Survey. Error bars represent 95% confidence intervals. Abbreviations: LDL, low-density lipoprotein.

Among participants who did not have diabetes, hypertension, or high LDL cholesterol and were not obese, 7.1% had MetS (Figure 1). Women with hypertension (77.0%) were more likely to have MetS compared with men with hypertension (41.2%) (*t* = 4.07, *P* < .001), and women with high LDL cholesterol (56.3%) were more likely to have MetS compared with men with high LDL cholesterol (40.0%) (*t* = 2.19, *P* = .03).

**Table:** 

**Comorbid condition**	**Overall**		**Men**		**Women**	

	**%**	**95% CI**	**%**	**95% CI**	**%**	**95% CI**
**Diabetes**	69.4	57.1-79.5	70.2	56.8-80.9	66.4	49.7-79.8
**Hypertension**	55.9	46.2-65.2	41.2	29.3-54.2	77.0	63.9-86.4
**High LDL cholesterol**	47.0	39.2-55.0	40.0	30.6-50.2	56.3	44.5-67.3
**Obesity**	49.7	44.0-55.5	47.4	38.7-56.2	50.7	42.6-58.7
**No comorbidity**	7.1	4.7-10.7	6.2	3.3-11.5	7.5	4.3-12.7

### Multiple logistic regression models

**Figure 2.**
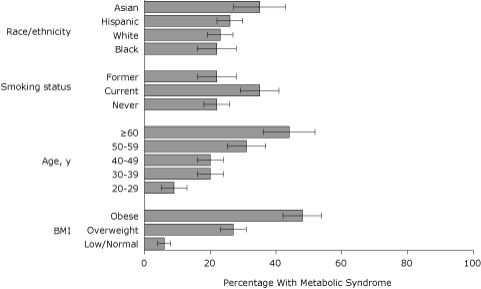
Adjusted prevalence (predicted marginals) of metabolic syndrome by race/ethnicity, smoking status, age, and body mass index (BMI), 2004 New York City Health and Nutrition Examination Survey. BMI (kg/m^2^) categories were defined as underweight (low), <18.5; normal, 18.5-24.9; overweight, 25.0-29.9; and obese, ≥30. Each estimate is adjusted for sex, alcohol use, the interaction between sex and alcohol use, and all other variables in figure. Error bars represent 95% confidence intervals.

**Figure 3.**
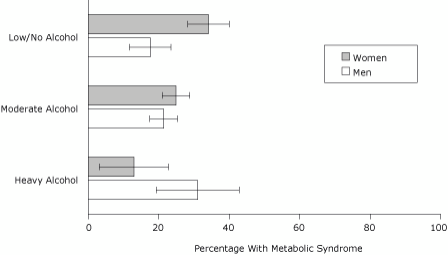
Adjusted prevalence (predicted marginals) of metabolic syndrome by sex and level of alcohol use, 2004 New York City Health and Nutrition Examination Survey. Alcohol use was defined as heavy (>2 drinks/day during the past year for men or >1 drink/day for women); moderate (less frequent use); or low/none (no drinking in the past year). Each estimate is adjusted for body mass index, age, smoking, and race/ethnicity. Error bars represent 95% confidence intervals.

In the final model, BMI and age remained positively associated with MetS (Figure 2). Prevalence was higher among current smokers than among never-smokers (AOR, 2.6; 95% CI, 1.8-3.8) and higher among Asians than among whites (AOR 2.4, 95% CI 1.4-4.1). Although prevalence of MetS was higher among men reporting heavy alcohol use compared with men reporting low or no alcohol use (AOR 2.8, 95% CI 1.1-7.6), it was lower among women who reported heavy or moderate alcohol use than among women who reported low or no alcohol use (AOR 0.2, 95% CI 0.1-0.6 and AOR 0.5, 95% CI 0.3-0.9, respectively) (Figure 3).

**Table:** 

**Characteristic**	**Adjusted Prevalence, %**
**BMI, kg/m^2^**	
**Low/normal (<24.9)**	6
**Overweight (25.0-29.9)**	27
**Obese (≥30)**	48
**Age (years)**	
**20-29**	9
**30-39**	20
**40-49**	20
**50-59**	31
**≥60**	44
**Smoking status**	
**Never**	22
**Current**	35
**Former**	22
**Race/ethnicity**	
**Black**	22
**White**	23
**Hispanic**	26
**Asian**	35

**Table:** 

**Level of alcohol use**	**Adjusted prevalence among men %**	**Adjusted prevalence among women %**
**Heavy**	31	13
**Moderate**	21	25
**Low/none**	18	34

### Exploration of the relationship between alcohol use and sex

In both sexes, median waist circumference was larger in participants reporting low or no alcohol use than in participants reporting moderate or heavy use; the difference was more pronounced among women than men (Table 3). Median triglycerides, HDL cholesterol, and fasting blood glucose levels were better among women reporting heavy alcohol use than among women reporting low or no alcohol use. Among men, however, the lowest triglyceride levels were observed in men reporting moderate alcohol use. No clear patterns emerged for other metabolic measurements.

## Discussion

MetS affects more than one-fourth of NYC adults, accounting for more than 1.5 million people at greater risk for CVD-related morbidity and mortality. Among people with MetS, the most common combination of syndrome components (abdominal obesity, elevated fasting blood glucose, and elevated blood pressure) may be associated with a particularly high risk of CVD (13). As in previous studies (7,8,14), we observed a steady increase in MetS prevalence with age and with BMI, suggesting that the scope of this problem is likely to grow as NYC's population ages and if obesity becomes more prevalent. Our finding that most NYC adults without MetS have 1 or more metabolic abnormalities related to the syndrome is further evidence of the widespread risk of developing MetS.

In keeping with previous reports of lower rates of hypertension, hypercholesterolemia, and obesity in NYC than in the United States overall (15), the age-adjusted prevalence of MetS in NYC HANES 2004 (26.7%) was lower than the national prevalence (34.0%) observed in NHANES 2003-2006 (6). Except for low HDL cholesterol (NYC, 27.8% vs national, 24.7%), the prevalence of each MetS abnormality was lower in the NYC population than in the national population (abdominal obesity, 47.0% vs 52.8%; elevated triglycerides, 22.5% vs 31.2%; elevated blood pressure, 31.3% vs 39.5%; and elevated fasting blood glucose, 35.7% vs 38.6%). Among women, except for abdominal obesity (NYC, 64.3% vs national, 60.7%), the prevalence of each MetS abnormality was also lower among NYC populations than in the national sample: elevated triglycerides (21.0% vs 26.5%), elevated blood pressure (30.4% vs 35.2%), and elevated fasting blood glucose (24.9% vs 31.3%) (6). Differences between men in the 2 samples were more pronounced: abdominal obesity (26.8% vs 44.8%), elevated triglycerides (24.5% vs 35.6%), elevated blood pressure (33.0% vs 43.4%), and elevated fasting blood glucose (43.1% vs 45.8%) (6).

The prevalence of MetS was 7.2 percentage points higher among women than among men in our study. Although recent NHANES studies did not find a difference between sexes, the NHANES 1988-1994 and NHANES 1999-2000 found a marked increase in prevalence among women, driven primarily by increases in blood pressure, waist circumference, and triglyceride levels (14). We found that abdominal obesity was common among women in the general NYC population (64.3%) and was a syndrome-defining characteristic for more than half of women with MetS, compared with 33.0% of men. As recommendations for prevention and treatment of MetS are developed, a special focus on preventing abdominal obesity among NYC women may be warranted.

We also observed differences in the relationship between alcohol use and MetS between sexes; level of alcohol use was inversely associated with MetS among women but increased the likelihood of MetS among men. Differences between sexes were also identified in the general NYC HANES population when we examined individual metabolic measures according to level of alcohol use. These data contrast with NHANES III data, which showed both an inverse relationship between level of alcohol use and MetS risk for both sexes (16) and a protective effect among women only (7). Other studies have also presented conflicting results on whether alcohol use has a protective or detrimental effect on MetS risk (17,18). These discrepancies may have resulted because of differences in how the studies defined alcohol use. Alternatively, because alcohol use has a positive impact on certain MetS components (19) but a variable effect on others (16), the relationship between alcohol use and MetS may differ according to the metabolic risk profile of the group being studied. Our results may be due to confounders we could not control. Regardless, additional studies adequately powered to elucidate the complex relationship between sex, alcohol use, and the components of MetS are needed.

The diversity of our sample enabled us to examine MetS among Asians, which has not been examined nationally. The age-adjusted MetS prevalence among Asians in NYC (9) was similar to the prevalence among whites, but Asians had a higher prevalence of MetS after adjustment for other factors. Other studies have reported higher rates of diabetes and cardiovascular disease among Asians compared with whites (20,21). These data suggest an opportunity for targeted prevention efforts, of particular importance for other areas with large Asian American communities.

Despite a close link between obesity and MetS, the prevalence of MetS was not negligible (6.5%) among normal-weight participants. Some studies have suggested that such people are already "metabolically obese" and may be susceptible to the negative effects of future weight gain (7,22) and have thus advocated aggressive dietary and exercise interventions (22). No guidelines exist for identifying and treating MetS in normal-weight people, but if this group is overlooked, so too would be their elevated risk of CVD.

Our study had several limitations, including the small sample size, which prevented extensive examination of risk factors for MetS among demographic subgroups. We may have underestimated MetS prevalence because we excluded from analysis people with incomplete data; these people were more likely to be low-income or overweight. We did not assess associations between MetS and dietary and exercise patterns because NYC HANES gathered limited data on these topics. Finally, that the ATP III definition of MetS may not have uniform validity among age, sex, and racial/ethnic groups (5,7) is of particular relevance in diverse settings such as NYC. Nonetheless, the definition we chose has been used extensively, enabling comparisons among studies.

Pending the development of recommendations for MetS screening and management, clinicians may wish to consider screening high-risk groups to help motivate people to pursue lifestyle changes that can mitigate or reverse MetS, including weight loss and increased physical activity (5). In particular, an opportunity exists for CVD risk modification among New Yorkers with MetS but without comorbid conditions and among New Yorkers with MetS who smoke. Detection of MetS and integration of MetS screening into routine practice may improve as electronic medical records become more widely available (23).

Meanwhile, the widespread prevalence of MetS and its components suggests that population-level interventions are needed to address CVD risk. NYC has already taken several steps, including initiatives to encourage calorie labeling in chain restaurants, decrease levels of sodium in foods, eliminate use of trans fats in food establishments, increase the availability of fresh produce, and create an environment conducive to physical activity. Our findings may enable further tailoring of broad-scale prevention policies to the NYC population and will provide a benchmark by which such efforts may be measured.

## Figures and Tables

**Table 1:** Age-Adjusted Prevalence of Metabolic Abnormalities Among New York City Adults Aged 20 Years or Older, 2004 New York City Health and Nutrition Examination Survey

**Prevalence or number**	Total, % (95% CI) (n = 1,263)	Men, % (95% CI) (n = 539)	Women, % (95% CI) (n = 724)
**Prevalence of abnormality**			
Abdominal obesity^a^^,^ b	47.0 (43.9-50.2)	26.8 (22.7-31.4)	64.3 (60.5-67.9)
Elevated triglyceride level^c^	22.5 (19.8-25.5)	24.5 (20.7-28.8)	21.0 (17.5-25.0)
Low HDL cholesterol level^d^	27.8 (24.8-31.0)	24.2 (20.5-28.3)	30.9 (26.8-35.2)
Elevated blood pressure^e^	31.3 (28.7-34.0)	33.0 (29.2-37.1)	30.4 (27.5-33.6)
Elevated fasting blood glucose^b^^,^ f	35.7 (32.7-38.8)	43.1 (38.3-48.1)	29.4 (25.7-33.4)
**Number of metabolic abnormalities**			
0	24.7 (22.4-27.2)	27.1 (24.0-30.4)	22.6 (19.7-25.7)
1	27.7 (25.1-30.6)	29.1 (24.7-33.8)	26.4 (23.2-29.9)
2	20.9 (18.3-23.7)	20.9 (17.3-25.0)	20.9 (17.6-24.8)
3	16.0 (13.6-18.7)	14.0 (10.9-17.7)	17.8 (14.6-21.6)
4	6.5 (5.1-8.2)	5.9 (4.1-8.5)	7.0 (5.2-9.4)
5	4.1 (2.9-5.9)	3.0 (1.7-5.4)	5.2 (3.4-8.0)

Abbreviations: CI, confidence interval; HDL, high-density lipoprotein.

a Abdominal obesity is defined as waist circumference ≥102 cm in men or ≥88 cm in women.

b Significant difference (by *t*- test) between men and women. We conducted comparisons between men and women only for specific metabolic abnormalities, not for number of metabolic abnormalities.

c Elevated triglyceride level is defined as a fasting triglyceride level ≥150 mg/dL.

d Low HDL is defined as <40 mg/dL in men or <50 mg/dL in women.

e Elevated blood pressure is defined as systolic ≥130 mmHg and/or diastolic ≥85 mmHg and/or use of medications for hypertension at time of survey.

f Elevated blood glucose is defined as fasting glucose ≥100 mg/dL or use of insulin or oral hypoglycemic medication or both at time of survey.

**Table 2:** Age-Adjusted Prevalence of Metabolic Syndrome Among New York City Adults Aged 20 Years or Older, by Selected Characteristics, 2004 New York City Health and Nutrition Examination Survey

Characteristic	Total (n = 1,263)			Men (n = 539)			Women (n = 724)	
	No.	% (95% CI)		No.	% (95% CI)		No.	% (95% CI)
Total	1,263	26.7 (23.7-29.8)		539	22.9 (19.1-27.2)		724	30.1 (26.3-34.1)
**Age group, y**								
20-29 [Reference]	325	7.3 (4.9-10.7)		150	8.4 (4.7-14.5)		175	6.3 (3.7-10.5)
30-39	301	18.4 (14.1-23.6)^a^		128	19.4 (13.1-27.7)^b^		173	17.5 (12.2-24.5)^c^
40-49	288	21.0 (16.8-25.8)^a^		113	18.0 (12.1-26.0)^b^		175	23.3 (17.6-30.1)^a^
50-59	194	34.7 (27.7-42.4)^a^		73	26.2 (16.8-38.3)^c^		121	40.6 (31.7-50.2)^a^
≥60	155	49.2 (39.6-58.9)^a^		75	40.2 (28.1-53.7)^a^		80	58.8 (45.5-71.0)^a^
**Race/ethnicity^d^**								
White [Reference]	366	21.8 (17.3-27.1)		175	20.1 (15.0-26.5)		191	23.5 (17.2-31.4)
Black	272	28.5 (23.5-34.0)		108	24.0 (15.8-34.6)		164	33.4 (26.9-40.6)^b^
Hispanic	452	33.9 (28.5-39.8)^c^		177	27.4 (20.3-35.9)		275	38.2 (31.9-44.8)^c^
Asian	156	23.0 (15.9-32.0)		72	23.6 (14.2-36.6)		84	22.4 (13.2-35.4)
**Education^d^**								
<HS degree/GED	369	35.3 (30.1-40.8)^a^		150	31.0 (23.7-39.3)^b^		219	38.4 (31.6-45.7)^c^
HS degree/GED	245	26.3 (20.0-33.7)		113	19.4 (11.3-31.2)		132	33.1 (24.9-42.4)
>HS degree/GED [Reference]	647	22.3 (18.6-26.5)		274	20.4 (15.7-26.0)		373	25.1 (20.2-30.8)
**Country of birth^d^**								
United States [Reference]	614	27.1 (23.2-31.4)		260	24.1 (19.0-30.1)		354	29.4 (24.1-35.4)
Other	648	26.1 (22.4-30.1)		278	20.5 (15.6-26.6)		370	31.4 (26.9-36.3)
**Income, $^d^**								
<25,000	507	28.2 (24.1-32.6)		195	21.5 (16.1-28.2)		312	32.8 (27.3-38.9)
25,000-49,999	308	26.5 (20.7-33.3)		142	24.2 (16.8-33.6)		166	28.7 (21.1-37.7)
≥50,000 [Reference]	398	24.5 (19.3-30.5)		187	22.6 (16.2-30.6)		211	26.6 (19.5-35.2)
**Insurance coverage^d^**								
Private [Reference]	596	24.5 (20.6-28.9)		257	21.6 (16.6-27.7)		339	27.3 (22.0-33.4)
Government	330	32.6 (27.3-38.)^b^		109	25.6 (17.7-35.4)		221	38.0 (31.5-44.9)^b^
Uninsured	332	24.4 (18.5-31.4)		169	24.1 (16.4-33.9)		163	24.8 (16.4-35.6)
**Routine source of care^d^**								
Present [Reference]	874	27.3 (23.9-31.0)		342	22.8 (18.3-28.1)		532	31.0 (26.8-35.5)
Absent	388	25.1 (18.5-33.0)		197	22.3 (13.9-33.8)		191	28.7 (19.9-39.6)
**Smoking**								
Never [Reference]	726	25.2 (21.6-29.2)		269	19.2 (14.0-25.6)		457	29.6 (25.7-34.0)
Current	319	33.9 (28.5-39.9)^b^		160	28.6 (20.6-38.1)		159	39.6 (31.0-49.0)
Former	218	23.4 (18.1-29.7)		110	23.8 (17.1-32.0)		108	23.1 (15.8-32.5)
**Alcohol use^d^**								
Heavy	99	27.5 (18.4-39.0)		49^e^	36.6 (24.9-50.0)		50^e^	12.0 (5.2-25.4)^a^
Moderate	775	23.7 (20.0-27.8)^b^		378	21.9 (17.3-27.3)		397	26.1 (20.8-32.2)^c^
Low or none [Reference]	384	32.4 (27.5-37.8)		110	21.8 (14.5-31.2)		274	38.1 (32.5-44.0)
**Body mass index, kg/m^2^ d**								
Underweight (<18.5)	28^e^	0		10^e^	0		18^e^	0
Normal weight (18.5-24.9) [Reference]	464	6.5 (4.1-10.3)		182	6.3 (3.4-11.6)		282	6.8 (3.6-12.5)
Overweight (25-30)	432	28.2 (23.8-33.2)^a^		212	20.1 (14.7-26.9)^a^		220	38.7 (33.5-44.1)^a^
Obese (≥30)	316	49.7 (44.0-55.5)^a^		122	47.4 (38.7-56.2)^a^		194	50.7 (42.6-58.7)^a^
**Menopausal status**								
Premenopausal [Reference]	NA	NA	NA	NA	NA	NA	530	29.8 (22.1-38.9)
Postmenopausal	NA	NA	NA	NA	NA	NA	183	26.7 (22.2-31.7)

Abbreviations: CI, confidence interval; GED, general educational development; HS, high school; NA, not applicable.

a *P* value < .001. Comparisons were made by *t* test to the reference group in each category.

b *P* value < .05. Comparisons were made by *t* test to the reference group in each category.

c *P* value < .01. Comparisons were made by *t* test to the reference group in each category.

d Totals do not equal to 1,263 because of missing data.

e Relative standard error was 30% or more or denominator was 50 or fewer (or both). We considered such estimates to be unreliable.

**Table 3:** Metabolic Measurements According to Alcohol Use and Sex Among New York City Adults Aged 20 years or Older, 2004 New York City Health and Nutrition Examination Survey

**Metabolic Measure**	**Alcohol Use^a^**	Men, Median (95% CI)	Women, Median (95% CI)
Waist circumference, cm	Heavy^b^	91.6 (88.1-96.4)	87.0 (83.0-93.0)
	Moderate	94.1 (92.6-95.9)	91.5 (90.1-94.2)
	Low/none	95.5 (93.5-98.6)	95.5 (93.4-98.6)
Triglycerides, mg/dL	Heavy^b^	123 (104-169)	84 (72-96)
	Moderate	106 (100-115)	91 (84-96)
	Low/none	117 (110-124)	110 (93-121)
HDL cholesterol, mg/dL	Heavy^b^	46 (42-53)	65 (59-70)
	Moderate	47 (45-49)	58 (55-60)
	Low/none	43 (41-45)	53 (51-55)
Systolic blood pressure, mmHg	Heavy^b^	117 (112-122)	106 (104-112)
	Moderate	116 (113-118)	107 (105-109)
	Low/none	118 (115-121)	112 (110-115)
Diastolic blood pressure, mmHg	Heavy^b^	73 (71-76)	68 (66-72)
	Moderate	73 (71-74)	68 (67-69)
	Low/none	73 (70-76)	68 (66-70)
Fasting blood glucose, mg/dL	Heavy^b^	100 (92-111)	88 (86-93)
	Moderate	96 (95-98)	92 (90-93)
	Low/none	99 (94-103)	95 (94-97)

Abbreviations: CI, confidence interval; HDL, high-density lipoprotein.

a Alcohol use defined as heavy (>2 drinks/d during the past year for men or >1 drink/d for women); moderate (less frequent use); or low/none (no drinking in the past year).

b Denominator was 50 or fewer for both men and women who reported heavy alcohol use. We considered such estimates to be unreliable.

## References

[1] Wilson PWF, Kannel WB, Silbershatz H, D'Agostino RB (1999). Clustering of metabolic factors and coronary heart disease. Arch Intern Med.

[2] Isomaa B, Almgren P, Tuomi T, Forsen B, Lahti K, Nissen M (2001). Cardiovascular morbidity and mortality associated with the metabolic syndrome. Diabetes Care.

[3] Malik S, Wong ND, Franklin SS, Kamath TV, L'Italien GJ, Pio JR (2004). Impact of the metabolic syndrome on mortality from coronary heart disease, cardiovascular disease, and all causes in United States adults. Circulation.

[4] Kahn R (2007). Metabolic syndrome: is it a syndrome? Does it matter?. Circulation.

[5] Grundy SM, Cleeman JI, Daniels SR, Donato KA, Eckel RH, Franklin BA (2005). Diagnosis and management of the metabolic syndrome: an American Heart Association/National Heart, Lung, and Blood Institute scientific statement. Circulation.

[6] Ervin RB (2009). Prevalence of metabolic syndrome among adults 20 years of age and over, by sex, age, race and ethnicity, and body mass index: United States, 2003-2006. National Health Statistics Reports; no 13.

[7] Park YW, Zhu S, Palaniappan L, Heshka S, Carnethon MR, Heymsfield SB (2003). The metabolic syndrome: prevalence and associated risk factor findings in the US population from the Third National Health and Nutrition Examination Survey, 1988-1994. Arch Intern Med.

[8] Ford ES, Giles WH, Dietz WH (2002). Prevalence of the metabolic syndrome among US adults: findings from the Third National Health and Nutrition Examination Survey. JAMA.

[9] Rajpathak SN, Gupta LS, Waddell EN, Upadhyay UD, Wildman RP, Kaplan R (2010). Elevated risk of type 2 diabetes and metabolic syndrome among Asians and south Asians: results from the 2004 New York City HANES. Ethn Dis.

[10] Thorpe LE, Gwynn RC, Mandel-Ricci J, Roberts S, Tsoi B, Berman L (2006). Study design and participation rates of the New York City Health and Nutrition Evaluation Survey, 2004. Prev Chronic Dis.

[11] Executive summary, and treatment (2001). Executive summary of the third report of the National Cholesterol Education Program (NCEP) expert panel on detection, evaluation, and treatment of high blood cholesterol in adults (Adult Treatment Panel III). Expert Panel on Detection, Evaluation, and Treatment of High Blood Cholesterol in Adults. JAMA.

[12] Upadhyay UD, Waddell EN, Young S, Kerker BD, Berger M, Matte T (2010). Prevalence, awareness, treatment, and control of high LDL cholesterol in New York City, 2004. Prev Chronic Dis.

[13] Franco OH, Massaro JM, Civil J, Cobain MR, O'Malley B, D'Agostino RB (2009). Trajectories of entering the metabolic syndrome: the Framingham heart study. Circulation.

[14] Ford ES, Giles WH, Mokdad AH (2004). Increasing prevalence of the metabolic syndrome among US adults. Diabetes Care.

[15] Gwynn RC, Garg RK, Kerker BD, Frieden TR, Thorpe LE (2009). Contributions of a local health examination survey to the surveillance of chronic and infectious diseases in New York City. Am J Public Health.

[16] Freiberg M, Cabral HJ, Heeren TC, Vasan RS, Ellison RC (2004). Alcohol consumption and prevalence of the metabolic syndrome in the US: A cross-sectional analysis of the data from the Third National Health and Nutrition Examination Survey. Diabetes Care.

[17] Djousse L, Ellison C, Beiser A, Scaramucci A, D'Agostino RB, Wolf PA (2002). Alcohol consumption and risk of ischemic stroke: the Framingham study. Stroke.

[18] Yoon YS, Oh SW, Baik HW, Park HS, Kim WY (2004). Alcohol consumption and the metabolic syndrome in Korean adults: the 1998 Korean National Health and Nutrition Examination Survey. Am J Clin Nutr.

[19] Gaziano JM, Buring JE, Breslow JL, Goldhaber SZ, Rosner B, VanDenburgh M (1993). Moderate alcohol intake, increased levels of high-density lipoprotein and its subfractions, and decreased risk of myocardial infarction. N Engl J Med.

[20] McNeely MJ, Boyko EJ (2004). Type 2 diabetes prevalence in Asian Americans: results of a national health survey. Diabetes Care.

[21] Palaniappan L, Wang Y, Fortmann SP (2004). Coronary heart disease mortality for six ethnic groups in California, 1990-2000. Ann Epidemiol.

[22] Ruderman NB, Schneider SH, Berchtold P (1981). The "metabolically-obese," normal-weight individual. Am J Clin Nutr.

[23] Hivert MF, Grant RW, Shrader P, Meigs JB (2009). Identifying primary care patients at risk for future diabetes and cardiovascular disease using electronic health records. BMC Health Serv Res.

